# Efficacy of an occluding toothpaste on dentinal hypersensitivity over 14 days

**DOI:** 10.1038/s41405-021-00076-3

**Published:** 2021-07-13

**Authors:** Jonathan E. Creeth, Chhaju Goyal, Jimmy Qaqish, Robert Maclure, Jonathan S. Holt

**Affiliations:** 1 GSK Consumer Healthcare, Weybridge, Surrey UK; 2 All Sum Research Centre Ltd, Mississauga, ON Canada; 3 Intertek Clinical Research Services, Ellesmere Port, Cheshire UK

**Keywords:** Tooth erosion, Gingival recession

## Abstract

**Objectives:**

To evaluate a 0.454% stannous fluoride/5% sodium tripolyphosphate (STP) toothpaste’s ability to provide relief from dentinal hypersensitivity (DH) applied using ‘focused brushing.’

**Materials and methods:**

In two randomised, examiner-blind, parallel-group studies, a SnF_2_/STP toothpaste was applied by brushing two selected sensitive teeth before 1 min whole-mouth brushing, compared to 1 min whole-mouth brushing only, with a negative control toothpaste. DH was assessed via evaporative (air) (Schiff scale) and tactile (Yeaple probe) stimuli after 7 and 14 d of twice-daily brushing.

**Results:**

In total, 141 (Study 1)/142 (Study 2) participants were randomised. In Study 1, the test treatment significantly reduced DH at 7/14 d versus baseline (7/14 d Schiff difference: −0.74 [−0.84,−0.65]/−1.39 [−1.54,−1.23]; tactile: 6.00 [4.88,7.13]/15.30 [13.34,17.26]); whereas the Control treatment did not (7/14 d Schiff difference −0.03 [−0.13,0.06]/−0.10 [−0.25,0.06]; tactile: 0.77 [−0.36,1.90]/0.77 [−1.20,2.74]). Differences between Test and Control were statistically significant (*p* < 0.0001 all cases). In Study 2, both treatments reduced DH compared to baseline by both measures, but there were no significant between-treatment differences. Toothpastes were generally well-tolerated.

**Discussion and conclusions:**

Previous studies and Study 1 support SnF_2_/5% STP toothpaste efficacy; Study 2 results may have been influenced by placebo/Hawthorne effects. DH study design needs to, where possible, negate such effects.

## Introduction

Dentine can become exposed due to gum recession or enamel loss, leading to patent dentine tubules.^1,2^ According to the hydrodynamic theory, various stimuli––thermal, tactile, chemical, osmotic or evaporative, such as a hot drink or cold air––lead to movement of fluid in exposed dentinal tubules that stimulates nerve fibres in the pulp and causes a short, sharp pain.^3–5^ Up to 84% of the population report experiencing DH at some point in their life.^6^ While for some this pain is described as an occasional annoyance, for others it is a near-constant problem.^7^

Treatment of DH with daily-use oral care products generally focuses on one of two approaches. Nerve depolarisation, which blocks the pain response, can be achieved using potassium ions.^8–10^ These generally require at least 14 d before a benefit is established.^11^ Tubule occlusion––whereby exposed ends of dentine tubules are physically blocked to isolate the nerve from external stimuli––can be accomplished with the use of ingredients such as bioglasses,^12,13^ arginine plus particulate calcium^14,15^ and metal ions such as strontium^16,17^ and stannous.^17–19^

Stannous fluoride (SnF_2_) has long been used in anti-DH toothpaste formulations. Clinical studies demonstrate the efficacy of SnF_2_ toothpastes across a wide time-scale from after a single brushing to up to many months’ use.^18–24^ Recently, studies have investigated how to reduce the time frame between using a SnF_2_ toothpaste and obtaining DH relief. Two routes have been explored: (i) the effect of application technique, specifically the effect of direct application prior to brushing, and (ii) the effect of formulation, specifically via optimisation of the base formulation polymer system.

Studies investigating direct application to sensitive areas prior to whole-mouth brushing have shown an advantage of brushing the sensitive teeth first for a defined period––christened ‘focused brushing’^22–26^––or by massaging the toothpaste into the affected teeth with a finger.^16,27,28^ Focused brushing studies have used a protocol in which the test product with an anti-DH ingredient has been applied directly to sensitive teeth prior to whole-mouth brushing, whereas the negative control product has been applied only by whole-mouth brushing.^22,23^ The protocol is therefore testing a regimen of test product/application technique versus control product/application technique. This approach attempts to mimic a ‘real-world’ scenario where individuals suffering from DH, who purchase a specific anti-DH toothpaste to treat it, would brush their sensitive teeth first, to ensure good contact of the anti-DH ingredients in the product with those sensitive areas. In contrast, those purchasing a conventional toothpaste would have no reason to apply it directly to their sensitive teeth (even if they suffered from DH), so would continue to brush normally.

The use of SnF_2_ as an anti-DH agent in a daily-use dentifrice brings formulation challenges. The stannous ion needs protecting from oxidation and/or hydrolysis during product storage prior to use. This can be achieved by formulating into an anhydrous, glycerine base. In addition, there are concerns about the staining potential of stannous ion-based toothpastes that have led to the inclusion of stain prevention and/or chemical cleaning agents, such as polyphosphates, into toothpaste formulations.^25,26^ The chelating agent sodium tripolyphosphate (STP) can be added to a SnF_2_ toothpaste without compromising DH efficacy^17^ and has been shown to prevent stain build-up over 8 weeks.^29^

In vitro studies of such SnF_2_ formulations have provided insight into the mode of action. On exposure to the aqueous oral environment, tubule openings at the dentine surface are occluded by precipitates of stannous hydroxides and oxides together with particulates from the toothpaste and saliva-derived proteins and ions, leading to a reduction in tubular fluid flow.^19,29–32^ Studies involving optimising the formulation polymer system in this type of anhydrous base have shown that increasing the level of carbomer polymer can increase the rate of deposition/retention of stannous ion in vitro, while maintaining longer-term performance.^33^

While two separate studies were undertaken, they both aimed to evaluate the ability of an experimental non-aqueous 0.454% SnF_2_/5% STP toothpaste with elevated carbomer level, applied by the focused brushing technique, to provide relief from DH, as elicited by evaporative and tactile stimuli after 7 and 14-d of use, compared to a regular family fluoride toothpaste without known anti-DH or specific whitening agents.

## Methods

These two 14-day studies followed a randomised, examiner-blind, two treatment-arm, parallel design and were stratified by maximum baseline Schiff sensitivity score and, for Study 2, by clinical site. Study 1 was conducted at a research facility in Canada; Study 2 was conducted at two sites (Ellesmere Port and Manchester, UK) of a UK-based research facility. Both were conducted in accordance with the Declaration of Helsinki and approved by independent research ethics committees before initiation (Study 1: Veritas IRB Inc. Reference 205201-2016-GSK-1; Study 2: North West––Liverpool Central Research Ethics Committee; Reference 16/NW/0065). These studies are registered at ClinicalTrials.org (Study 1: NCT02773758, Study 2: NCT02705716). Anonymised individual participant data and study documents can be requested for further research from www.clinicalstudydatarequest.com.

### Participants

Both studies enrolled healthy participants aged 18–65 y with no clinically significant or relevant abnormalities on oral examination. Participants were required to have a self-reported history of DH lasting more than 6 mo but not more than 10 y and ≥20 natural teeth. At screening, eligible participants had at least two accessible teeth (incisors, canines or premolars) with signs of erosion, abrasion and/or facial/cervical gingival recession (EAR), a Modified Gingival Index^34^ score of 0, a clinical mobility score ≤1 and a positive response to a qualifying evaporative (air) assessment. At baseline (Day 0), eligible participants had a minimum of two accessible, non-adjacent teeth with signs of sensitivity, determined by a qualifying tactile stimulus threshold of ≤20 g and a Schiff sensitivity score ≥2.^9^

General exclusion criteria included pregnancy; breastfeeding; any known/suspected allergy or intolerance to study materials; participation in another clinical study or receipt of an investigational drug within 30 d or participation in a tooth-desensitising treatment study within 8 weeks prior to screening; a chronic debilitating disease that could affect study outcome; a xerostomia-causing condition/medication; daily use of medication that could affect pain perception; current use of antibiotics or use within 2 weeks prior to baseline visit.

General oral exclusions included tongue/lip piercings; dental implants; gross periodontal disease; exposed dentine with deep, defective or facial restorations; dental prophylaxis within 4 weeks, desensitising treatment or tooth bleaching within 8 weeks, scaling or root planing within 3 mo, periodontal disease treatment within 12 mo of screening. Specific dentition exclusions for test teeth included: current/recent caries, or reported treatment of decay, within 12 mo of screening; teeth used as abutments for fixed/removable partial dentures; full crowns or veneers; orthodontic bands; cracked enamel or sensitive teeth with contributing aetiologies other than EAR; sensitive teeth not expected, in the investigator’s opinion, to respond to treatment with an over-the-counter toothpaste.

### Procedures

At the screening visit, each participant provided written informed consent to participate in the study before their demographic characteristics, medical history and use of concomitant medications were recorded. An oral soft tissue (OST) examination was then conducted. Each participant’s dentition was assessed sequentially for evidence of EAR; gingival health status using the Modified Gingival Index;^34^ tooth mobility using a modification of the Miller scale;^35^ and sensitivity to an evaporative (air) stimulus (where a ‘yes’ response from the participant indicated sensitivity).

Eligible participants were supplied with a local market standard fluoride toothpaste (Study 1: Crest^®^ Cavity Protection with 1000 ppm fluoride as sodium fluoride; Procter & Gamble, Cincinnati, OH, US; Canadian marketed product; Study 2: Signal^®^ Family Protection with 1000 ppm fluoride as sodium monofluorophosphate [SMFP]; Unilever, London, UK; UK marketed product) and a toothbrush (Aquafresh^®^ Clean Control [Everyday Clean], GSK Consumer Healthcare, Brentford, UK [GSKCH]) to use twice daily for 4–8 weeks between screening and baseline visits (acclimatisation period). While the lead-in toothpaste was different between the studies, differences between them with regard to relief of DH are not expected.^36^ First use of the toothpaste was carried out under supervision at the study site. Each use was recorded in a supplied diary.

At the baseline visit (Day 0), ongoing eligibility was assessed, any adverse events, incidents and changes to concomitant medications were recorded and compliance with the use of acclimatisation toothpaste was evaluated. Following an OST examination, hypersensitivity of the clinically eligible teeth identified at screening was evaluated by the participant’s response to two separate, independent stimuli, in accordance with consensus guidelines in DH studies.^37^ The first DH assessment involved a tactile stimulus, administered by a constant-pressure Yeaple probe.^38^ After at least 5 min (to ensure recovery from the tactile stimulus), teeth with a tactile threshold ≤20 g were evaluated for hypersensitivity to an evaporative (air) stimulus, assessed using the Schiff Sensitivity Scale.^9^ These two assessment techniques measure response to quite different types of dentine stimulus, to increase robustness of any observations of treatment effects. The Schiff examiner selected two non-adjacent hypersensitive teeth from those that met the qualifying assessments to be evaluated for the remainder of the study.

Before the study visits, participants refrained from all oral hygiene procedures and from taking analgesics for at least 8 h, from eating and drinking for at least 4 h and from excessive alcohol consumption for 24 h. Small sips of room temperature water were permitted for taking medication/relieving thirst if needed within 4 h before the visits but not within 1 h. During the study, participants could not use any dental products other than those provided, nor any products for treating sensitive teeth. Use of dental floss was permitted for the removal of impacted food. Participants were to delay having any non-emergency dental treatment until after study completion.

Eligible participants were randomised to one of two treatment regimens according to a randomisation schedule provided by the study sponsor. Randomisation numbers were assigned in ascending numerical order as each participant was determined eligible. Randomisation was stratified by maximum baseline Schiff sensitivity score (either 2 or 3) of the two selected test teeth. The Test toothpaste contained 0.454% SnF_2_ (1100 ppm fluoride) and 5% STP. Participants applied a full ribbon of this toothpaste to a dry toothbrush then brushed each of the selected test teeth, followed by brushing the whole mouth for at least 60 s. The Control toothpaste contained 0.76% SMFP (1000 ppm fluoride) (Colgate^®^ Cavity Protection; Colgate-Palmolive, New York, NY, US; USA marketed product). Participants applied a full ribbon of this toothpaste to a dry toothbrush then brushed the whole mouth for at least 60 s.

Both groups followed their brushing regimen twice a day (morning and evening) for 14 d. First use of the study toothpaste was carried out under supervision at the study site. Compliance with use of the study toothpaste was assessed by review of the participant-completed diary cards. At each visit, participants underwent an OST examination before any clinical assessment of sensitivity. The dental examiners, study statistician, data management staff and other employees of the sponsor who could have influenced study outcomes were blinded to toothpaste allocation.

All study product tubes were overwrapped with white vinyl to blind participants to toothpaste assignment. The details of each brushing regimen were not discussed, i.e. that only the Test toothpaste included focused brushing, so participants were not made aware that the Control toothpaste regimen did not include focused brushing; hence they should not have known their group assignment.

### Assessments

Clinical assessments of tooth sensitivity were made after 7 and 14 d of toothpaste use. As for the baseline assessments, two independent, stimulus-based clinical measures were used to assess DH, in accordance with consensus guidelines,^37^

Firstly, a tactile stimulus was administered using a constant-pressure (Yeaple) probe,^38^ which permitted application of a known force to the tooth surface. The greater the tactile threshold, the less sensitive the tooth. Testing began at a pressure of 10 g and was increased by 10 g with each successive challenge until either two consecutive ‘yes’ responses (with ‘yes’ indicating the stimulus caused pain or discomfort) were at the same pressure setting (recorded as the tactile threshold in grams) or the maximum force was reached. At baseline, maximum force was 20 g; at subsequent visits, it was 80 g.

Secondly, after a minimum 5-min recovery period, evaporative (air) sensitivity was assessed by directing a jet of air from a triple air dental syringe onto the exposed dentine surface from a distance of ≈1 cm, with the test tooth surface isolated to prevent adjacent teeth or surrounding soft tissue being exposed to the stimulus.^9^ The examiner’s assessment of the participant’s response was recorded on the Schiff Sensitivity Scale (from 0 = participant does not respond to air stimulus, to 3 = participant responds to air stimulus, considers stimulus to be painful and requests discontinuation of the stimulus).

In Study 1, a single examiner performed all assessments. In Study 2, two examiners were employed across the two study centres, one who performed all the evaporative (air) assessments and one who performed all the tactile stimulus assessments.

### Safety

Spontaneously reported adverse events (AEs) and any abnormalities in the OST examination were recorded from the start of use with the acclimatisation toothpaste at the screening visit until 5 d after the last use of study toothpaste. Treatment-emergent AEs (TEAEs) were reported for the safety population, which included all randomised participants who received the study toothpaste.

### Data analysis

#### Sample size determination

Based on outcomes from previous sensitivity studies,^21^ for both Study 1 and 2 it was estimated that a sample of 60 participants per group would have an ≥90% power to detect a mean difference between the toothpastes of 0.35 units in Schiff sensitivity score (assuming a standard deviation [SD] of 0.5467) using a two-sided t-test of significance level 0.05.

#### Efficacy analyses

Efficacy analyses were performed on a modified intent-to-treat (mITT) population, defined as all randomised participants who provided at least one post-baseline assessment of efficacy. The efficacy variables for analysis were the change from baseline at Days 7 and 14 (based on the change in the mean value of the two selected test teeth) in evaporative (air) sensitivity (using the Schiff Sensitivity Scale) and tactile sensitivity (using the tactile threshold). The primary endpoint was the change from baseline in Schiff sensitivity score at 14 d. Change from baseline in Schiff sensitivity score was evaluated by analysis of covariance (ANCOVA), with treatment group and study site (Study 2 only) as factors, and baseline Schiff sensitivity score as a covariate. For tactile threshold, the mean baseline Schiff sensitivity score of the two selected test teeth was also included as a factor, with baseline tactile threshold score as a covariate. For all treatment groups, adjusted means and 95% confidence intervals (CIs) were calculated.

The ANCOVA model assumptions for the analyses of Schiff sensitivity score were investigated and considered to be satisfied for both studies. For the tactile threshold data, the residual normality assumption was in doubt for Study 1 and failed for Study 2, therefore, change in tactile threshold was also analysed by a non-parametric method (van Elteren test, adjusting for the maximum baseline Schiff Sensitivity scores). The results were compared with the ANCOVA results. For Study 1, the inferences from the two analyses were similar, therefore only the ANCOVA results are presented. For Study 2 only the van Elteren Test are presented for tactile threshold.

## Results

**Fig. 1:**
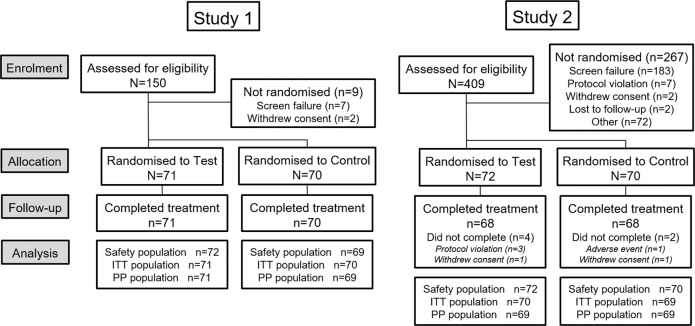
Participant disposition throughout the study.

For Study 1,150 participants were screened and 141 were randomised to treatment (Fig. 1). The first participant was enrolled on 25 January 2016, the last completed the study on 11 March 2016. Table 1 shows baseline characteristics by group. The majority of participants in the safety population were female (*n* = 105; 74.5%) and were white (*n* = 89; 63.1%); mean age was 47.9 y (SD: 10.39; range 18–64 y). Most participants were in Schiff strata ‘3’: 108 (76.6%).

**Table 1:** Summary of baseline characteristics (safety population).

	Study 1		Study 2	
Characteristic	Test (*n* = 72)	Control (*n* = 69)	Test (*n* = 72)	Control (*n* = 70)
Sex, *n* (%)				
Male	17 (23.6)	19 (17.5)	18 (25.0)	12 (17.1)
Female	55 (76.4)	50 (72.5)	54 (75.0)	58 (82.9)
Age, y				
Mean (SD)	47.7 (10.27)	48.0 (10.58)	39.6 (10.89)	39.9 (9.46)
Range	18–64	19–64	18–65	18–65
Race, *n* (%)				
White	40 (55.6)	49 (71.0)	59 (81.9)	60 (85.7)
Black/African American	15 (20.8)	10 (14.5)	7 (9.7)	7 (10.0)
Asian	16 (22.2)	10 (14.5)	3 (4.2)	3 (4.3)
Multiple	1 (1.4)	0	3 (4.2)	0
Schiff strata 2, *n* (%)	17 (23.6)	16 (23.2)	6 (8.3)	5 (7.1)
Schiff strata 3, *n* (%)	55 (76.4)	53 (76.8)	66 (91.7)	65 (92.9)

For Study 2,409 participants were screened and 142 were randomised to treatment (Site 1 207/76, respectively; Site 2 202/66, respectively) (Fig. 1). The first participant was enrolled on 8 March 2016, the last participant completed the study on 20 May 2016. Table 1 shows baseline characteristics by group. The majority of participants in the safety population were female (*n* = 112; 78.9%) and were white (*n* = 119; 83.8%); mean age was 39.7 y (SD: 10.18; range 18–65 y). Most participants were in Schiff strata ‘3’: 131 (92.3%).

The demographic characteristics of the treatment groups were similar across groups for the safety and ITT populations of both studies (Table 1).

### Efficacy

**Fig. 2:**
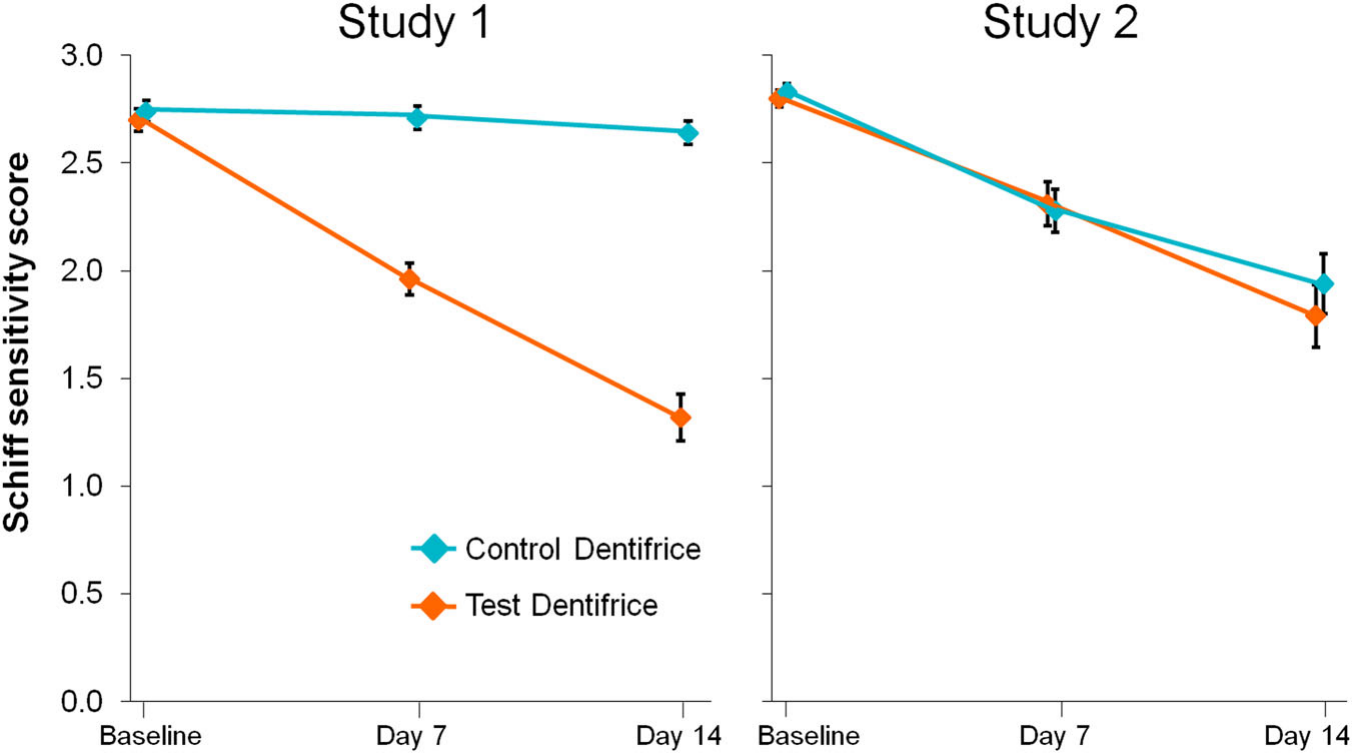
Effect of treatments on Schiff sensitivity scores. Values are adjusted means (±SE). Data are offset for clarity. Low values are favourable.

**Fig. 3:**
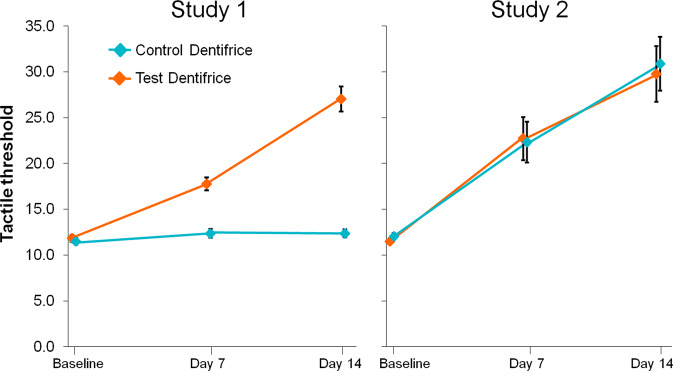
Effect of treatments on Tactile threshold scores. Values are adjusted means (±SE). Data are offset for clarity. Tactile threshold score range: 0–80 g. High values are favourable.

In Study 1, there was a statistically significant change from baseline with the Test toothpaste at Days 7 and 14 (both *p* < 0.0001), with decreases in Schiff sensitivity score of −0.74 (27.4%) and −1.39 (51.1%) respectively (Table 2, Fig. 2), and increases in tactile threshold score of 6.00 g (50.0%) and 15.30 g (128.6%) respectively (Table 2, Fig. 3). No significant differences were shown for the Control toothpaste on either measure. At both 7 and 14 d, the change from baseline was statistically significantly greater for the Test toothpaste compared to the Control for both Schiff sensitivity and tactile threshold scores (*p* < 0.0001 for all).

**Table 2:** Between-treatment comparisons for change in Schiff sensitivity score and tactile threshold (intent-to-treat population).

			Test*	Control*	Test vs Cont**
Schiff sensitivity score					
Study 1	BL	2.70 (0.051)		2.74 (0.051)	
	7 d	−0.74 (−0.84, −0.65) −27.4% *p* < 0.0001		−0.03 (−0.13, 0.06) −1.1% *p* = 0.5059	−0.71 (−0.85, −0.57) −26.4% *p* < 0.0001
	14 d	−1.39 (−1.54, −1.23) −51.1% *p* < 0.0001		−0.10 (−0.25, 0.06) −3.6% *p* = 0.2276	−1.29 (−1.51, 1.07) −49.2% *p* < 0.0001
Study 2	BL	2.80 (0.039)		2.83 (0.037)	
	7 d	−0.49 (−0.67, −0.30) −17.5% *p* < 0.0001		−0.54 (−0.73, −0.62) −19.4% *p* < 0.0001	0.06 (−0.21, 0.32) 2.2% *p* = 0.67699
	14 d	−1.01 (−1.27, −0.74) −36.1% *p* < 0.0001		−0.89 (−1.15, −0.62) −31.4% *p* < 0.0001	−0.12 (−0.50, 0.25) −6.2% *p* = 0.5191
Tactile threshold					
Study 1	BL	11.83 (0.378)		11.50 (0.358)	
	7 d	+6.00 (4.88, 7.13) 50.0% *p* < 0.0001		+0.77 (−0.36, 1.90) 7.5% *p* = 0.1807	5.23 (3.63, 6.83) 42.0% *p* < 0.0001
	14 d	+15.30 (13.34, 17.26) 128.6% *p* < 0.0001		+0.77 (−1.20, 2.74) 7.5% *p* = 0.4428	14.53 (11.75, 17.31) 116.8% *p* < 0.0001
Study 2	BL	11.50 (0.327)		12.03 (0.362)	
	7 d	+11.35 (6.91, 15.79) 97.5% *p* < 0.0001		+10.15 (5.68, 14.63) 85.5% *p* < 0.0001	1.19 (−5.12, 7.51) 5.5% *p* = 0.7093
	14 d	+18.58 (13.00, 24.15) 159.0% *p* < 0.0001		+18.56 (12.98, 24.13) 156.7% *p* < 0.0001	0.02 (−7.89, 7.92) 0.1% *p* = 0.9962

Baseline values are raw means, all other are adjusted means from ANCOVA model.

*BL* Baseline, *d* day.

*Change from baseline (95% confidence interval), percentage change, *p* value. Percentage change calculated as 100*[(Treatment–BL)/BL].

**Difference (95% confidence interval), percentage change, *p* value: first-named minus second-named group such that, for Schiff sensitivity score, a negative difference favours the first-named group or, for tactile threshold, a positive difference favours the first-named group. Percentage change calculated as: 100*[(overall mean BL value + Test adjusted change from baseline)−(overall mean BL value + Control adjusted change from baseline)/(overall mean BL value + Control adjusted change from baseline)].

In Study 2, there was also a statistically significant change from baseline with the Test toothpaste at Days 7 and 14 (both *p*<0.0001, with decreases in Schiff sensitivity score of −0.49 (17.5%) and −1.01 (36.1%) respectively (Table 2, Fig. 2), and increases in tactile threshold score of 11.35 g (97.5%) and 18.58 g (159.0%) respectively (Table 2, Fig. 3). For the Control toothpaste, at both time points statistically significant differences were also shown in both Schiff sensitivity score (decreases of −0.54 [19.4%] and −0.89 [31.4%] for 7 and 14 d respectively) and tactile threshold score (increases of 10.15 g [85.5%] and 18.56 g [156.7%] for 7 and 14 d respectively) (Table 2, *p* < 0.0001 for all). There were no significant differences between the Test and Control toothpastes for either measure, at either time point.

### Safety

In Study 1 there were three reported TEAEs, by three participants in the Test group, none of which was oral or considered treatment-related. All TEAEs were graded ‘mild’ and had resolved by the end of the study with no withdrawals due to a TEAE.

In Study 2, there were 19 TEAEs reported by 15 participants in the Test group, 30 TEAEs reported by 10 participants in the Control group. There were 10 oral TEAEs (eight participants) in the Test group, three oral TEAEs (three participants) in the Control group. TEAEs were mild or moderate in intensity and all but one (lost tooth restoration) had resolved by the end of the study. Only one TEAE was considered treatment-related: one incidence of mild oral mucosal exfoliation in the Test group. One TEAE led to participant withdrawal: root canal infection of moderate intensity in the Control group.

## Discussion

In the first of these two near-identically run studies (Study 1), a Test toothpaste containing 0.454% SnF_2_ and 5% STP was shown to significantly improve DH compared to baseline, using two separate measures, when applied using a ‘focused brushing’ regimen. These differences were clinically meaningful on both measures by 14 d of use according to criteria of Orchardson et al.,^11^ a reduction from baseline of at least 33%. The Control toothpaste, containing no known anti-DH ingredients, performed as would be expected, in that there were no significant changes from baseline, meaning the Test treatment was significantly more effective in the two separate DH measures at both time points. These between-treatment differences were clinically meaningful from 7 d, according to American Dental Association criteria of a difference between treatments of at least 20%.^39^

However, in Study 2, while a similar significant, clinically meaningful change from baseline was shown for the Test treatment on both measures, statistically significant changes were also shown for the Control treatment, with no significant between-treatment differences.

The fact that such a different result was observed between studies with a near-identical design raises issues somewhat endemic to pain studies, even of treatments with accepted efficacy. That is, why do studies sometimes fail to show the expected difference between test and negative control treatments? There have been many studies of SnF_2_ toothpastes that confirm their efficacy in decreasing DH,^18–24^ including those of 2 weeks’ duration, 3 d and even immediate use.^22–24^ Therefore, the efficacy of the SnF_2_ toothpaste is hard to question.^17^ These current results mirror those of Parkinson et al.^21^ who used a similar study design and closely related test and negative control toothpastes in three studies. While the test toothpaste was statistically significantly superior to the negative control by Day 14 in two of the studies, there were no such differences in the other trial. A substantial improvement in DH for the negative control appeared to be a key factor. In view of the existing body of evidence, therefore, the results of the current studies suggest that Study 2 was a ‘false negative,’ in which a true difference (as seen in Study 1) was, for some reason or reasons, not observed. Potential reasons for this outcome are discussed below.

Two factors are especially prevalent in pain studies such as this that use subjective assessments. One is the placebo effect––the response to a treatment of no known efficacy above that of not administering a treatment.^40^ The other is the Hawthorne effect––whereby behaviour changes occur due to the act of being observed in a study.^41^ DH studies are known to be prone to both effects.^42,43^ West et al.^43^ suggested a range of 20–60% of treatment effects are due to a placebo response in conventional DH studies. In the three studies detailed in Parkinson et al.,^21^ reductions from baseline for the negative control treatment ranged from 15–26% for Schiff sensitivity scores, with increases from baseline in tactile threshold scores ranging from 41–74%. Here, respective percentage changes at 8 weeks for the Control toothpaste were 31.4% and 156.7%, nearly identical to those with the Test toothpaste.

One of the few traits consistently found in those individuals liable to show a placebo response is a high baseline pain severity.^44–46^ This would suggest that the higher the degree of DH at screening, the greater the chance of improvement during the study, irrespective of treatment. This is of interest here because in Study 2 almost all the participants (overall 92.3%) had the highest Schiff sensitivity score of ‘3,’ compared to 76.6% of those in Study 1. While participants were stratified by baseline Schiff score to ensure an even number between treatments, this balances rather than reduces the problem, and it may simply be the case that differences between treatments are more apparent when the participant population has less severe initial DH than was prevalent in Study 2.

Another issue is the episodic nature of DH, which means that it may resolve without treatment.^43^ Hence, though care was taken to only include those who had experienced DH for at least 6 mo (though not necessarily in the test teeth), a natural resolution of DH could have been experienced by a sufficient proportion of participants in Study 2 to swamp product differences.

Treatment response may also be influenced by the Hawthorne effect. This arises because people tend to change their behaviour when they are being observed. An observational study of people with long term DH who used either an anti-DH or a non-anti-DH toothpaste, tracked them over 24 weeks and found improvements in both groups, suggesting that merely being in the study had brought about a change, despite participants knowing whether or not their toothpaste was targeting DH and not being assigned a toothpaste that was new to them.^47^

It is also known that those with DH may over-brush, thinking that the reason for experiencing DH is that they are not carrying out proper oral hygiene.^43,48^ Hence, changes in DH could be a response to being instructed on a gentler brushing regimen.^43^ In these current studies, while an explicit brushing style was not given, participants were told they could only brush their teeth for 1 min twice a day. If more of those in Study 2 than in Study 1 had been over-brushing, limiting their brushing may have reduced the amount of pain they experienced due to nerve stimulation by brushing action, regardless of toothpaste used. In addition, a reduction in brushing may have allowed greater undisturbed precipitation of calcium, phosphates and proteins from saliva onto the dentine surface, enhancing blockage of dentine tubules irrespective of dentifrice used .^43^

Responses to treatment have also been shown to vary according to how a healthcare professional interacts with the participant.^44^ The release of endorphins elicited by positive emotional and behavioural responses can lead to a reduction in pain response, regardless of whether active treatment is given.^49^ Furthermore, if interactions with the assessor are on a very personable level, this can lead to the participant wanting to ‘please’ the assessor by, for instance, responding that treatment has had a greater effect on pain than in fact it has.^43^ This encourages using a fixed ‘script’ for DH studies. One difference between the studies was that in Study 1, there was a single examiner, but in Study 2, there were two, one for each measure. While both approaches are acceptable, to either limit inter-examiner differences by only having one, or control for examiner error and bias by having two, it would be expected that if the reason for no treatment differences was due to examiner error in Study 2, this would only be reflected in only one of the measures, not both.

Benedetti et al.^41^ suggest that screening should also include an assessment of participant expectation and perceived treatment assignment to ascertain if this could contribute to the results. The differences in how participants were recruited between the studies could potentially have played a part in participant expectation. Differences in screening versus randomised participant numbers between study sites (Study 1: 150 screened, 141 randomised; Study 2: 409 screened, 142 randomised) were investigated and found to be due to differences in pre-screening criteria. In Study 1, participants were drawn from a heavily pre-screened database that had previously been registered, so there may have been a longer ‘wait-list’ period than in Study 2 where participants were recruited and screened in the same period. This shorter time between recruitment and study participation in Study 2 may have meant there were more participants than in Study 1 with an increased expectation of relief from DH due to study participation. This difference in recruitment approach is also believed to explain the high exclusion rate of participants at screening in Study 2, who were initially recruited based on a brief qualifying questionnaire with many failing at baseline due to the finding that they were using a banned medication or toothpaste (usually one that, while it may not have been explicitly labelled as such, did contain potential anti-DH ingredients) or that they did not have enough sensitivity at screening or baseline.

Having examined several of the factors that may have contributed to the differences shown between the two studies, it is of note that the design of these, and many other DH studies, included an acclimatisation period. This should have helped to overcome factors such as expectation of symptom relief and potential of spontaneous symptom relief, and acclimatised participants to being in a study and to possibly altering their brushing regimen. To account for any participants whose DH receded in this time, they were re-examined at baseline, with stricter criteria than screening, prior to dispensing of study treatment.

A final consideration as to why no differences were found in Study 2 is that the studies were only powered to provide a 90% chance of seeing a difference of the expected size as statistically significant, with the consequence of a one-in-ten chance that, even if the expected difference were observed, it would be found not statistically significant. This chance is in addition to natural biological variation in observed treatment difference, which will vary from the true difference from study to study.

Taken together, these experimental factors will combine to tend to reduce the degree of difference observed between an effective DH treatment and a negative control. It is, therefore, possible that the true benefit of the Test treatment is greater than that indicated by the study set.

Nevertheless, in terms of whether the evidence presented in this manuscript supports efficacy of the test product, we need also to consider the possibility that Study 1 showed an erroneously large difference between the products, i.e. it was a ‘false positive,’ and Study 2 was in fact the more accurate estimate of the true product difference. We believe this should be considered much less likely than the ‘false negative’ scenario described above, as the probability of the difference observed in study 1 not being real was below 1/10000, according to the statistical analysis. Randomisation and blinding were used to remove systematic bias that could have artificially exaggerated the true product difference.

In summary, studies such as those reported here are prone to a range of issues associated with subjective pain assessment. Although it cannot be stated with confidence from the data presented here in isolation that the Test SnF_2_ toothpaste treatment was superior to the Control toothpaste treatment in reducing DH, the authors’ conclusion is that the balance of evidence is in favour of efficacy of the SnF_2_ toothpaste treatment. This conclusion is based on the discussion above, showing that it is in practice much more likely that a ‘false negative’ has occurred in Study 2 than a ‘false positive’ in Study 1. This position is supported by data from a number of shorter- and longer-term studies clearly showing efficacy of SnF_2_ toothpastes against marketed control toothpastes, using similar clinical methodologies.
